# Soluble Glass and Its Applications

**Published:** 1872-04

**Authors:** 


### ARTICLE X.

### *Soluble Glass and its Applications.*

The recent epidemic of fires, if we may be allowed the expression, has in many cases called the attention of the public to this remarkable compound, and stimulated inquiry as to its uses and the methods of applying it. Although it has been known for many years, it has thus far failed to come into very general use, partly because the public have expected more from it than was reasonable, and have consequently been disappointed in practice; and partly because the extravagant praises of some of its friends have placed it in the same category as quack medicines and other articles which are said to cure all diseases and remove all difficulties, and hence are regarded by the more sensible portion of the community as not being good for anything. In reality, however, it does possess the most valuable properties, and may be used to great advantage under a great variety of circumstance.

Soluble glass is simply a variety of purely alkaline glass in which the alkali is in excess. Ordinary window-glass is a compound of silica with potash or soda, and in some cases lime; oxide of lead added to the compound of silica and potash or soda gives flint glass; Bohemian glass is a compound of silica, soda and lime; and the coarse glass used for bottles contains much more iron and some alumina, which is the base of clay. According to the quantity of alkali employed, the glass will be soluble or insoluble; it being understood that all glass is soluble to a certain extent. Old window-panes that have been exposed to the elements for years, are in general so corroded that their surfaces are no longer perfectly transparent; and common flint glass, when finely powdered, dissolves in water to such an extent that its presence can be detected by the least delicate reagents. But when the proportion of alkali is largely increased, and especially when the compound consists of pure alkali and pure silica, we obtain a glass which dissolves entirely in water, and which may be applied as an incombustible varnish to wooden articles, or used as a cement or as a coating for brick and stone. Soluble glass was first brought into practical use by Prof. Fuchs, of Munich, in Bavaria, in the year 1823, and hence is frequently known as Fuch's soluble glass. At first it was prepared by fusing ten parts of pearlashes, fifteen parts of powdered quartz, and one part of charcoal together, and pulverizing the mass, which was then added in small portions at a time to boiling water until the whole was dissolved. The solution was then evaporated to a jelly-like consistency, when it was ready for market. More recently it has been found that certain varieties of silica are soluble in a boiling solution of caustic soda; and also that, when the temperature of an alkaline solution is greatly increased, which may be done by boiling it in a close vessel under great pressure, flints and other hard varieties of silica dissolve rapidly. It is in this way, we believe, that Ransome prepares the soluble glass used in the manufacture of his famous artificial stone. It is therefore obvious, from a consideration of these methods, that soluble glass is readily prepared; and, as the materials are comparatively cheap, there is no reason why it should not come into very extensive use, provided it should prove really valuable in the arts.

The first notable application of soluble glass was to the theatre of Munich, where it was used for the purpose of preventing the recurrence of a fearful disaster by fire. Before trusting to its protective qualities, however, a test was made of its powers, and a small building coated with soluble glass was erected in one of the public squares, and attempts made to fire it at several points, by placing small heaps of light wood in contact with it and setting those heaps on fire. Of course, where the flames came in contact with the building, the wood of which it was made was charred, and to a certain extent destroyed. But in no case did the building itself take fire or burn; and the test was deemed so satisfactory that the theatre was immediately coated in such a way as to be made fireproof. Since that time, it has been applied in many cases, and always with success whenever the application was made with a moderate amount of skill. That it might be used extensively for preventing fires, and for adding to the durability of all wooden structures, is unquestionable; and therefore a few hints as to the best methods of using it may not be out of place. These hints we are enabled to give more readily, since the whole subject was carefully investigated by the celebrated French chemist, Dumas, who has, in his "Traite de Chimie applique aux Arts," detailed very fully the results at which he arrived, He found that, although soluble glass is of itself a good preservative from fire, it fulfils the object better when it is mixed with another incombustible body in powder. Clay, whiting, calcined bones, powdered glass, etc., may all be employed for the purpose, though it is difficult to decide which of them is the best. A mixture of clay and whiting appears to be better than either used separately.

Flint glass, and the crude soluble glass as it comes from the furnace, are excellent additions. The powdered soluble glass ought to be exposed to the air until it has attracted some moisture; after which, if it be mixed with the solution and applied to any body whatever, it will in a short time form a coating as hard as stone, which, if the glass be of good quality, is unalterable by exposure, and resists fire admirably. When soluble glass is used for rendering wood fireproof or indestructible, it is always well to apply, in the first place, a coating of the pure glass. The pores are in this way filled up; while, if we use a thick and paintlike mixture of the solution with some powder, the liquid does not penetrate beneath the surface, and much of the effect is lost. When properly prepared, soluble glass, after being dried by exposure to the air, suffers a change which renders it incapable of being washed off. The alkali not being completely neutralized in this form of glass, it is difficult to apply oil paint to woodwork that has been treated with it; but this objection might be remedied by treating the prepared surface, when dry, with a weak solution of acid.—*Am. Jour. Pharm.*

